# Case Report: Hodgkin Lymphoma and Refractory Systemic Lupus Erythematosus Unveil Activated Phosphoinositide 3-Kinase-δ Syndrome 2 in an Adult Patient

**DOI:** 10.3389/fped.2021.702546

**Published:** 2021-07-08

**Authors:** Francesca Conti, Arianna Catelli, Cristina Cifaldi, Lucia Leonardi, Rita Mulè, Marco Fusconi, Vittorio Stefoni, Maria Chiriaco, Beatrice Rivalta, Silvia Di Cesare, Gioacchino Schifino, Fabiana Sbrega, Gigliola Di Matteo, Simona Ferrari, Caterina Cancrini, Andrea Pession

**Affiliations:** ^1^Pediatric Unit, IRCCS Azienda Ospedaliero-Universitaria di Bologna, Bologna, Italy; ^2^Specialty School of Paediatrics - Alma Mater Studiorum, University of Bologna, Bologna, Italy; ^3^Department of Systems Medicine, University of Rome Tor Vergata, Rome, Italy; ^4^Academic Department of Pediatrics, Immune and Infectious Diseases Division, Research Unit of Primary Immunodeficiencies, Bambino Gesù Children's Hospital, IRCCS, Rome, Italy; ^5^Maternal, Infantile and Urological Sciences Department, Sapienza University of Rome, Rome, Italy; ^6^Rheumatology Unit, Azienda Ospedaliero-Universitaria di Bologna, Bologna, Italy; ^7^Institute of Hematology “L. e A. Seràgnoli”, University of Bologna, Bologna, Italy; ^8^Respiratory and Critical Care Unit - IRCCS Azienda Ospedaliero Universitaria di Bologna, Bologna, Italy; ^9^Department of Specialistic, Diagnostic and Experimental Medicine (DIMES), Alma Mater University, Bologna, Italy; ^10^Scuola di Specializzazione di Patologia Clinica e Biochimica Clinica, Università Alma Mater Studiorum, Bologna, Italy; ^11^U.O. Genetica Medica, IRCCS Azienda Ospedaliero-Universitaria di Bologna, Bologna, Italy

**Keywords:** lymphoma, refractory SLE, immunodeficiency, *PIK3R1*, PI3K signaling, APDS2, IFN-signature

## Abstract

**Introduction:** Activated phosphoinositide 3-kinase-δ syndrome 2 (APDS2) is a rare primary immune regulatory disorder caused by heterozygous gain of function mutation in the *PIK3R1* gene encoding PI3Kδ regulatory p85α subunit and resulting in PI3Kδ hyperactivation. Clinical features range from recurrent infections to manifestations of immune dysregulation like autoimmunity, inflammation, systemic lymphoproliferation, and increased risk of cancer. We describe a new dominant *PIK3R1* mutation causing APDS2 presenting with lymphoma and systemic refractory autoimmunity.

**Case Presentation:** A 30-year-old woman was referred to the Immunology Unit of our hospital for uncontrolled systemic lupus erythematosus, including chilblains lesions, systemic lymphoproliferation and IgA deficiency. At 19 years of age, she was diagnosed with Hodgkin's lymphoma. Subsequently, she presented systemic lupus erythematosus onset, with episodes of severe exacerbation, including autoimmune hemolytic anemia and pleuro-pericarditis. Initial clinical response to conventional treatments was reported. Immunological investigations performed during our first observation showed severe lymphopenia, IgA deficiency, elevated IgM with reduced IgG2 levels, and low vaccination antibody titers. Quantitative real-time polymerase chain reaction (PCR) assay for Cytomegalovirus and Epstein-Barr virus showed low viral loads for both viruses in serum. An increase of serum inflammatory markers highlighted persistent systemic hyperinflammation. The next-generation sequencing (NGS)-based gene panel tests for primary immunodeficiency showed a heterozygous A>G substitution in the splice acceptor site at c.1300-2 position of *PIK3R1*, leading to exon-skipping.

**Conclusion:** This case emphasizes the importance of suspecting primary immune regulatory disorders in young adults, predominantly showing a severe, aggressive, and refractory to treatment immune dysregulation phenotype, even in the absence of major infectious diseases at the onset. Different treatments can be promptly started, and a delayed diagnosis can highly impact the outcome. Targeted therapy against PI3Kδ pathway defect effectively improves drug-resistant autoimmunity, lymphoproliferation, and risk of progression to malignancy; eligible patients could benefit from its use even as a bridge therapy to transplantation, currently the only definitive curative treatment. Therefore, identifying genetic mutation and prompt targeted treatment are essential to control disease manifestations, prevent long-term sequelae, and enable curative HSCT in APDS2 patients.

## Introduction

Activated phosphoinositide 3-kinase-δ syndrome 2 (APDS2) is a rare inborn error of immunity (IEI) presenting with features of immune dysregulation, classified as a predominantly antibody deficiency with hypogammaglobulinemia (1). The underlying monogenic defect involves autosomal dominant splicing defect of *PIK3R1* gene, leading to skipping of exon 11 and loss of inhibitory function of the encoded phosphoinositide 3-kinase (PI3K) regulatory subunit (p85α), resulting in hyperactivation of PI3K signaling, clinically phenocopying activating mutations of the PI3K catalytic subunit (p110δ) (2). Immunological features include B cells lymphopenia and class-switch-recombination defects (CSR-D) with reduced class-switched memory B cells and increased transitional B cells. Instead, the T-cell profile is characterized by an inverted CD4/CD8 ratio and a higher level of senescent T cells (CD57+CD3+). Increased serum IgM is commonly described while normal or decreased IgA and IgG (especially IgG2 class) can be detected (3). APDS2 is characterized by heterogeneous clinical manifestations, severity, and age of onset. Repeated infections with typical and atypical microorganisms primarily affect upper and lower respiratory tracts inducing a persistent inflammatory reaction associated with progressive airway damage. Herpesvirus susceptibility can cause severe disease, including neoplastic transformation, and chronic cytomegalovirus (CMV), Epstein-Barr virus (EBV), or herpes simplex virus (HSV) viremia (4). The presence of autoimmunity, systemic lymphoproliferation, hyper inflammation, and increased risk of neoplastic transformation may underly an inborn error of immune regulation (5). Growth retardation, mild neurodevelopmental delay, facial dysmorphisms, and tonsillar hypertrophy are typically observed in APDS2 patients (6, 7). We report a new heterozygous mutation of *PIK3R1* associated with APDS2, observed for the first time in a young adult with predominant immune dysregulation manifestations at the diagnosis.

## Case Description

A 30-year-old woman was referred to the Immunology Unit for uncontrolled systemic lupus erythematosus and systemic lymphoproliferation and IgA deficiency. She was the only daughter from non-consanguineous parents of Romanian descent, with no family history of immunological disease. During her infancy, growth retardation and common upper respiratory tract infections were observed. She underwent adenotonsillectomy for hypertrophic tonsils with a notable improvement of infectious diseases. No previous hospitalizations were reported.

At the age of 19, she was diagnosed with stage IIIA Hodgkin's lymphoma. Chemotherapy led to partial resolution. Stable remission was subsequently achieved following autologous stem cell transplantation.

Shortly afterward, she presented with polyarthritis, polyserositis, and chilblains, associated with leukopenia, antinuclear antibodies positivity, anti-double-stranded DNA antibodies positivity, and complement C3 reduction, leading to the diagnosis of systemic lupus erythematosus (SLE). Complete response was achieved after 6 months of standard therapy. Indeed, autoimmune hemolytic anemia and immune-mediated thrombocytopenia occurred 1 year after SLE onset. The patient was then treated with hydroxychloroquine and short periods of low-dose prednisone until the end of 2018.

**Figure 1:**
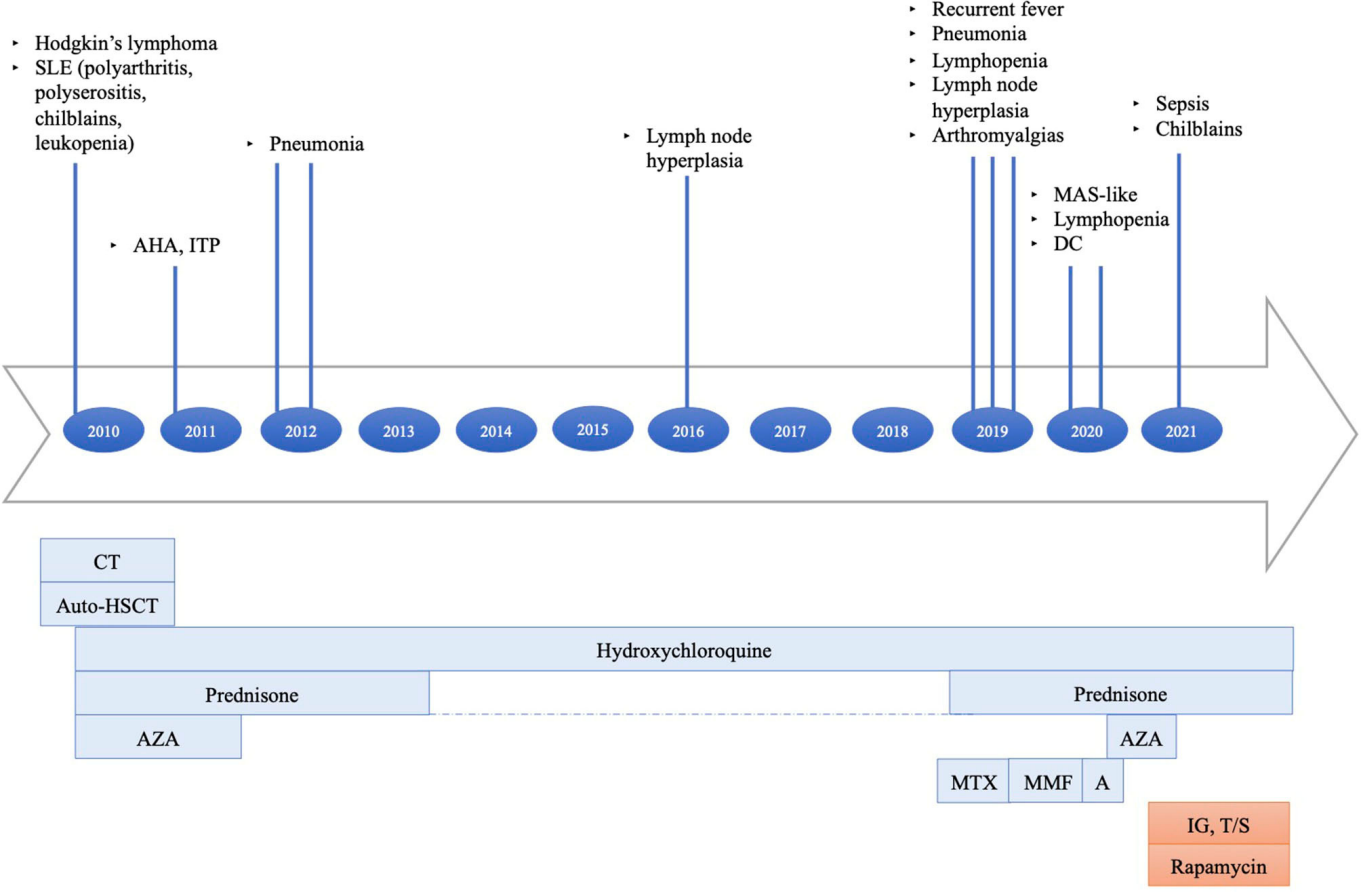
Principal clinical events and therapeutic strategies timeline: (1) Hodgkin's lymphoma treated with six cycles of doxorubicin, bleomycin, vinblastine, dacarbazine (ABVD), two cycles of ifosfamide, epirubicin, and etoposide (IEV); under stable remission after HSCT. (2) AHA, ITP treated with steroids. (3) SLE treated with hydroxychloroquine, azathioprine, and prednisone at onset, then with hydroxychloroquine and short periods of low-dose prednisone until the end of 2018. (4) Recurrent episodes of fever, diffuse arthralgias, myalgias, respiratory infections, MAS-like, chilblain lupus persisted despite treatment trials with high-dose steroids, methotrexate, mycophenolate mofetil, and anakinra. The last therapeutic attempt with azathioprine was interrupted because of side effects. SLE, systemic lupus erythematosus; AHA, autoimmune hemolytic anemia; ITP, immune-mediated thrombocytopenia; MAS, macrophage activation syndrome; DC, dilative cardiopathy; CT, chemotherapy; auto-HSCT, autologous stem cell transplantation; AZA, azathioprine; MTX, methotrexate; MMF, mycophenolate mofetil; A, anakinra; IG, immunoglobulin; T/S, trimethoprim/sulfamethoxazole.

Since February 2019, she has frequently presented with persistent lymphopenia, recurrent episodes of fever, chilblain lupus, diffuse arthralgias, myalgias, and respiratory infections, including sinusitis and pneumonia. A macrophage activation syndrome-like systemic inflammation was also reported. Disease manifestations were poorly responsive to therapeutic attempts (Figure 1).

**Figure 2:**
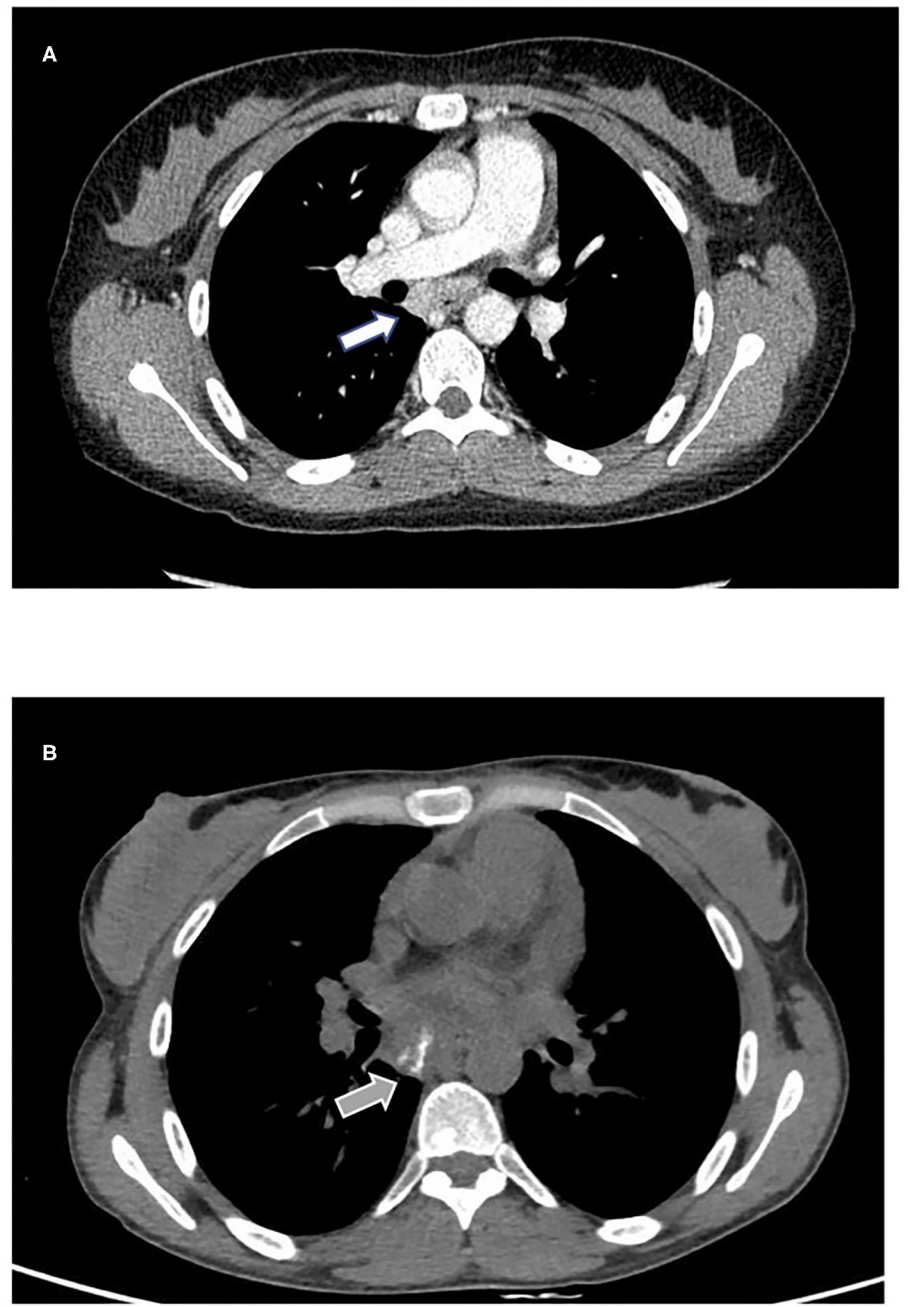
Mediastinal involvement. **(A)** Contrast enhancing subcarinal lymphadenopathy detected with CT scan performed in 2019 (white arrow). **(B)** One year follow up shows increased volume and intranodal calcification (gray arrow). No parenchymal abnormalities were found.

Lung high-resolution computed tomography (HRCT) was performed, revealing altered ventilation of the superior right lobe, middle lobe, lingula, and left lobes. Two solid nodules of 5 millimeters were evident at the superior left lobe. Mediastinal and right bronchial lymphadenopathies showed a growing pattern, and diffuse lymphoproliferation was documented at consecutive HRCTs (Figure 2). Positron emission tomography (PET) was performed to rule out the hypothesis of a malignancy relapse and highlighted diffuse lymphoproliferation, with involvement of lateral cervical, axillary, thoracic, gastric, iliac, and lower limb regions. Axillary lymph node biopsy excluded a lymphoma. Abdominal ultrasound and esophagogastroduodenoscopy also excluded lymphoproliferative disorders. Echocardiography documented left ventricular dilatation of unknown etiology. During our first observation, the patient presented with reduced body weight and height, hepatosplenomegaly, diffuse lymphadenopathies, malar rash, polyarthritis affecting small joints of the hands, and acral chilblains. Blood tests showed severe lymphopenia, normochromic normocytic anemia, thrombocytosis, the elevation of inflammatory markers, low complement C3 levels, and positive antinuclear antibodies. Immunological findings included IgA deficiency, increased IgM levels, and normal IgG with a reduction of IgG2 subclass. Serum IFN-I signature was significantly increased despite steroid therapy, as shown by the overexpression of six interferon-stimulated genes (IFI27, IFI44L, IFIT1, ISG15, RSAD2, SIGLEC1) in peripheral blood (Supplementary Figure 1). Chronic low-level CMV and EBV viremia were detected. Vaccination antibody titers were present for hepatitis B, pertussis, and rubella, alongside the absent response to diphtheria, tetanus toxoid, measles, and mumps. Cytofluorimetric immunophenotyping of peripheral blood lymphocytes revealed B-cell (CD19+) reduction in absolute and percentage count, with a poor subset of switched memory B cells (CD19+IgD-CD27+). At T cells analysis, we observed decreased absolute numbers of both helper (CD4+) and cytotoxic (CD8+) subpopulations and inverted CD4+/CD8+ ratio (Table 1).

**Table 1:** Immunological findings in the patient.

	**Patient**	**Reference range (10^**3**^ cells/μL)**
White blood cells, 10^3^ cells/μL	2.95	3.60–10.50
Neutrophils, 10^3^ cells/μL	2.29	1.50–7.70
Lymphocytes, 10^3^ cells/μL	0.381	1.20–4.10
CD3+ (PAN T), %(10^3^ cells/μL)	83% (0.316)	0.78–3.0
CD3+/α+β, %(10^3^ cells/μL)	99% (0.312)	0.6–3.3
CD3/γ+δ+, %(10^3^ cells/μL)	<1% (<0.003)	0.025–0.2
CD3+CD4-CD8-, %(10^3^ cells/μL)	<1% (<0.003)	0.0069–0.074
CD4, %(10^3^ cells/μL)	30% (0.094)	0.5–2.0
CD4+CD45 RA+ (Naïve), %(10^3^ cells/μL)	45% (0.042)	0.1–2.3
CD4+CD45 RA-CCR7+ (Central memory), %(10^3^ cells/μL)	48% (0.045)	0.18–1.1
CD4+CD45 RA-CCR7- (Effector memory), %(10^3^ cells/μL)	7% (0.006)	0.013–0.22
CD4+CD45 RA+CCR7- (Terminal effector memory), %(10^3^ cells/μL)	<1% (<0.0009)	0.000098–0.068
CD4+CD127-+CCR7+CD25++ (Regulatory), % (10^3^ cells/μL)	<1% (<0.0009)	0.025–0.18
CD8, %(10^3^ cells/μL)	52% (0.164)	0.2–1.2
CD8+CD45 RA+ (Naïve), %(10^3^ cells/μL)	45% (0.073)	0.016–1.0
CD8+CD45 RA-CCR7+ (Central memory), %(10^3^ cells/μL)	1% (0.001)	0.0047–0.12
CD8+CD45 RA-CCR7- (Effector memory), %(10^3^ cells/μL)	17% (0.028)	0.04–0.64
CD8+CD45 RA+CCR7- (Terminal effector memory), % (10^3^ cells/μL)	37% (0.060)	0.025–0.28
CD4/CD8 ratio	0.58	1.00–2.70
CD56+16+CD3- (NK), %(10^3^ cells/μL)	13% (0.049)	0.10–1.2
CD19 (PAN B), %(10^3^ cells/μL)	2.7% (0.010)	0.064–0.82
CD19+IgD+CD27- (B naïve), %(10^3^ cells/μL)	64% (0.006)	0.028–0.55
CD19+IgD+CD27+ (B memory), %(10^3^ cells/μL)	16.5% (0.001)	0.0039–0.17
CD19+IgD-CD27+ (switched B memory), %(10^3^ cells/μL)	2.7% (0.0002)	0.0045–0.13
CD19+CD21+CD38- (B CD21+low), %(10^3^ cells/μL)	1% (0.0001)	0.0017–0.049
CD19+IgM++CD38++ (B transitional), %(10^3^ cells/μL)	<1% (<0.0001)	0.0006–0.10
CD19+IgM-+CD38++ (B plasmablast), %(10^3^ cells/μL)	<1% (<0.0001)	0.0007–0.020
IgM, g/L	4.12	0.40–2.30
IgA, g/L	0.08	0.70–4.00
IgG, g/L	14.92	7.00–16.00
IgG1, g/L	11.71	3.82–9.28
IgG2, g/L	0.41	2.41–7.00
IgG3, g/L	0.80	0.22–1.76
IgG4, g/L	0.06	0.04–0.86

*Normal values for T cell subsets from Schatorjé et al. (8), and B cell subsets from Schatorje et al. (9); serum immunoglobulin concentrations from Aksu et al. (10)*.

**Figure 3:**
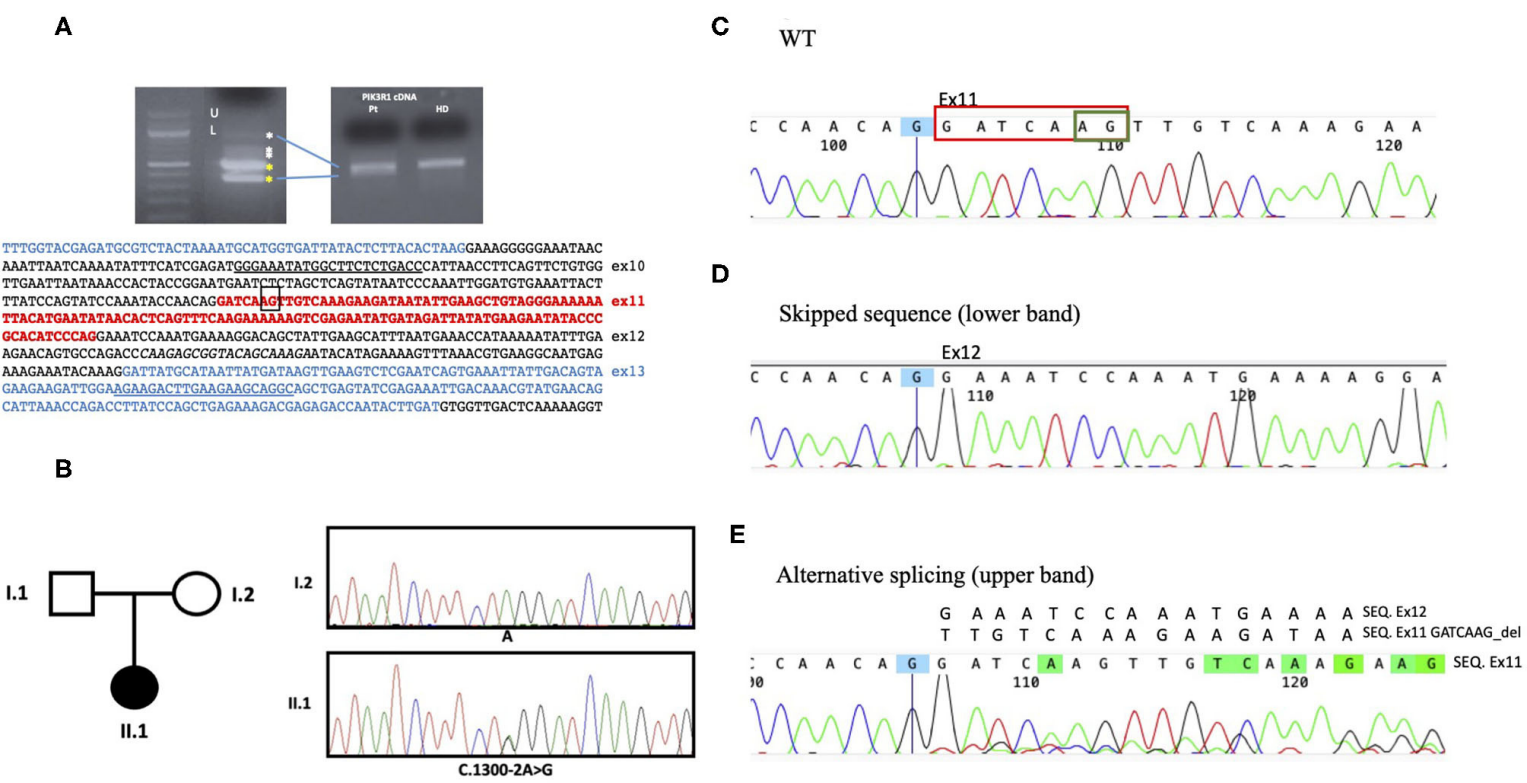
Genetic analysis. **(A)** By cDNA analysis, two main abnormal splicing products were demonstrated (U as upper and L as lower band). The PIK3R1 cDNA sequence of interest is indicated below. **(B)** Sanger sequencing electropherograms showing the PIK3R1 heterozygous point mutation c.1300-2A>G at the splice acceptor site present in the proband (II.1) and not in the mother (I.2). Blood sample from the father was not available **(C)** The wt sequence shows the correctly spliced transcript including exon11. **(D)** The intronic mutation c.1300-2A>G upstream exon11, abolished normal RNA splicing, resulting in either complete skipping of the exon 11 or in the usage of a cryptic acceptor exonic site, circled in green in the WT sequence. **(E)** The activation of this alternative splice site leads to removal of 7 nucleotides in exon 11 (circled in red in the WT sequence) causing the overlapping sequences in E.

The coexistence of persistent lymphoproliferation, the onset of Hodgkin's lymphoma, and uncontrolled SLE at a young age, together with the clinical and laboratory presentation, raised the suspicion of a congenital disorder of the immune system. Next-generation sequencing (NGS) panel for primary immunodeficiencies highlighted a heterozygous mutation in the *PIK3R1* gene in the exon 11 acceptor splice site (NM_181523.2:c.1300-2 A&gt; G). The variant was reported twice in ClinVar as likely pathogenic but, to our knowledge, has never been described before in the literature. Further tests confirmed that the newly described mutation results in the skipping of exon 11 and other minor splicing products of p85α, leading to the diagnosis of APDS2 (Figure 3).

Treatment focused on supportive therapy: subcutaneous immunoglobulin replacement was started. Moreover, to prevent opportunistic infections due to the severe CD4+ lymphopenia, antibiotic prophylaxis with trimethoprim/sulfamethoxazole was begun. At the same time, given the severity of the concomitant immune dysregulation manifestations, a combination therapy with immunosuppressive drugs was proposed. According to previously described APDS2-cohorts and ESID APDS registry (11), the patient agreed to start off-label treatment with rapamycin, which proved efficacy on non-malignant lymphoproliferation, one of the most detrimental and refractory clinical manifestations that she presented. Furthermore, the presence of chilblain lupus in our patient is likely explained by the high serum IFN-I signature. Literature reports demonstrate a potential link between PI3K-AKT-mTOR and type I IFN signaling pathways (12, 13), suggesting that our therapeutic strategy could also benefit from the use of JAK inhibitors like ruxolitinib or baricitinib. Clinical and immunological follow-up has been planned to monitor drug-related adverse events and long-term response, and to evaluate the need of new therapeutic perspectives in the future.

## Discussion

We describe the case of a 30-year woman affected by APDS 2 due to a new splice site mutation in the known hotspot in the *PIK3R1* gene encoding the regulatory subunit (p85α PI3Kδ). A recent systematic review on APDS showed hotspot mutation causing APDS2 with 79% frequency (6). As expected, this pathogenic variant causes the deletion of exon 11, resulting in a shortened p85α protein dominantly responsible for hyperactivated PI3Kδ signaling in T and B lymphocytes. Furthermore, disrupted p85α protein is responsible for extra-hematopoietic manifestations. The patient's clinical features reported here retrospectively analyzed after genetic diagnosis are similar to those described in the systematic review by Jamee et al.: short stature, tonsillar hypertrophy, hematologic malignancy, refractory autoimmunity, systemic lymphoproliferation, respiratory infections, and increased serum IgM levels (6). Interestingly, the case reported here differs from the majority of APDS2 patients described so far in term of the age of disease onset, that was at 19 years, significantly later than cases reported in the literature. This feature reflects that our patient lacked a notable infectious phenotype that generally represents the first alert symptoms leading to immunological investigations during the pediatric age.

Hodgkin's lymphoma and SLE were diagnosed at 19 years and treated as a separated entity for 10 years. The first immunological evaluation was conducted at the age of 30 after the worsening of clinical conditions, onset of systemic lymphoproliferation, recurrent infections, and in consideration of the multi-refractory to treatment SLE also associated with chilblain lupus. The median age of presentation of cancer and autoimmunity is in line with the data reported on the APDS2 cohort by Jamee et al. (6). However, the lack of previous suggestive medical history and the separate management of the two conditions led to a diagnostic delay of about 10 years.

Familial chilblain lupus is a monogenic form of cutaneous lupus erythematosus caused by loss-of-function mutations in the nucleases TREX1 or SAMHD1 and gain-of-function mutations in STING. To our knowledge, chilblains lesions are not described in the literature as a feature of the APDS2. For the first-time, we report chilblain lupus presumably explained by constitutive type I IFN activation in the case described. Recently, Langan Pai et al. described an association between type I IFN response and mTOR activation in PBMCs from TAFRO subtype of idiopathic multicentric Castleman disease, thus showing the link/interplay between PI3K-AKT-mTOR and type I IFN signaling pathway (12). Furthermore, a crucial role for the PI3K-AKT signaling pathway in Aicardi-Goutières Syndrome due to *SAMHD1* deficiency has been described (13). Our finding may expand the genetic spectrum of type I IFN-dependent disorders and suggests that JAK inhibition may be of therapeutic value in addition to PI3K-mTOR inhibitors treatments in APDS2 patients (14). Deeper studies on APDS patients are needed to confirm this hypothesis linking inborn errors of PI3K pathway to aberrant type I IFN response.

Our case illustrates the importance of awareness in adult non-immunological units about the APDS2 red flags in order to avoid significant diagnosis delays associated with increased morbidity and mortality of these patients. Lymphoma can be the onset symptom of APDS, and the median age of presentation reported is 16.5 years, corresponding to the age of transition care to adult medicine. Rheumatological disorders also generally occur during adolescence or in young adulthood, and all the refractory, relapsing, or unexplained severe cases should be investigated to exclude an underlying IEI. Immunological alterations are heterogeneous and are described in about 70% of APDS patients. Therefore, a first-level immunological workup, including lymphocyte subpopulations and serum immunoglobulin concentration, is recommended in patients with refractory autoimmunity or hematological malignancies associated with symptoms of immune dysregulation or red flags for a IEI, especially before starting oncological treatment protocol or immunosuppressive drugs. Indeed, prompt targeted treatment has a crucial role in controlling disease manifestations, preventing long-term sequelae, and enabling curative HSCT in APDS2 patients.

## Data Availability Statement

The original contributions presented in the study are included in the article/Supplementary Materials, further inquiries can be directed to the corresponding author/s.

## Ethics Statement

Ethical review and approval was not required for the study on human participants in accordance with the local legislation and institutional requirements. The patients/participants provided their written informed consent to participate in this study.

## Author Contributions

FC and AC conceptualized the article, participated to visualization and prepared the original draft. CCi, GDM, MC, and SDC participated to investigation, performing the immunological experiments and to the formal analysis of the article. LL, RM, MF, VS, BR, GS, and FS participated to data curation. CCa and AP participated to the validation of the article. SF participated to investigation, performing the genetic analysis and the formal analysis of the article. FC, CCa, and AP participated to project administration, supervised the work, reviewed and edited the article. All the authors have read and agreed to the published version of the manuscript.

## Conflict of Interest

The authors declare that the research was conducted in the absence of any commercial or financial relationships that could be construed as a potential conflict of interest.

## Supplementary Material

Supplementary Figure 1 Relative quantification of gene expression of six interferon-stimulated genes in peripheral blood. Serum IFN-I signature was increased as shown by **(A)** gene score and **(B)** interferon score. Interferon signature was obtained as published with modifications [*J Clin Immunol*. (2019) 39:476–85. doi: 10.1007/s10875-019-00645-0].
