# Corrigendum: Case Report: Hodgkin Lymphoma and Refractory Systemic Lupus Erythematosus Unveil Activated Phosphoinositide 3-Kinase-δ Syndrome 2 in an Adult Patient

**DOI:** 10.3389/fped.2021.757229

**Published:** 2021-09-17

**Authors:** Francesca Conti, Arianna Catelli, Cristina Cifaldi, Lucia Leonardi, Rita Mulè, Marco Fusconi, Vittorio Stefoni, Maria Chiriaco, Beatrice Rivalta, Silvia Di Cesare, Gioacchino Schifino, Fabiana Sbrega, Gigliola Di Matteo, Simona Ferrari, Caterina Cancrini, Andrea Pession

**Affiliations:** ^1^Pediatric Unit, IRCCS Azienda Ospedaliero-Universitaria di Bologna, Bologna, Italy; ^2^Specialty School of Paediatrics - Alma Mater Studiorum, University of Bologna, Bologna, Italy; ^3^Department of Systems Medicine, University of Rome Tor Vergata, Rome, Italy; ^4^Academic Department of Pediatrics, Immune and Infectious Diseases Division, Research Unit of Primary Immunodeficiencies, Bambino Gesù Children's Hospital, IRCCS, Rome, Italy; ^5^Maternal, Infantile and Urological Sciences Department, Sapienza University of Rome, Rome, Italy; ^6^Rheumatology Unit, Azienda Ospedaliero-Universitaria di Bologna, Bologna, Italy; ^7^Institute of Hematology “L. e A. Seràgnoli”, University of Bologna, Bologna, Italy; ^8^Respiratory and Critical Care Unit - IRCCS Azienda Ospedaliero Universitaria di Bologna, Bologna, Italy; ^9^Department of Specialistic, Diagnostic and Experimental Medicine (DIMES), Alma Mater University, Bologna, Italy; ^10^Scuola di Specializzazione di Patologia Clinica e Biochimica Clinica, Università Alma Mater Studiorum, Bologna, Italy; ^11^U.O. Genetica Medica, IRCCS Azienda Ospedaliero-Universitaria di Bologna, Bologna, Italy

**Keywords:** lymphoma, refractory SLE, immunodeficiency, *PIK3R1*, PI3K signaling, APDS2, IFN-signature

In the published article, there was an error in **affiliations 1, 4, 8, 11**. Instead of “Istituti di Ricovero e Cura a Carattere Scientifico,” it should be “IRCCS.”

In the published article, there was an error in **affiliations 1 and 11**. In **affiliation 1**, instead of “Pediatric Unit, Istituti di Ricovero e Cura a Carattere Scientifico Azienda Ospedaliero-Universitaria di Bologna, University of Bologna, Bologna, Italy”, it should be “Pediatric Unit, IRCCS Azienda Ospedaliero-Universitaria di Bologna, Bologna, Italy”.

In **affiliation 11**, instead of “Medical Genetics Unit, Istituti di Ricovero e Cura a Carattere Scientifico S.Orsola University Hospital, Bologna, Italy”, it should be “U.O. Genetica Medica, IRCCS Azienda Ospedaliero-Universitaria di Bologna, Bologna, Italy”.

The authors apologize for this error and state that this does not change the scientific conclusions of the article in any way. The original article has been updated.

## Publisher's Note

All claims expressed in this article are solely those of the authors and do not necessarily represent those of their affiliated organizations, or those of the publisher, the editors and the reviewers. Any product that may be evaluated in this article, or claim that may be made by its manufacturer, is not guaranteed or endorsed by the publisher.

