# Supplementary figures and images for: Case Report: Hodgkin Lymphoma and Refractory Systemic Lupus Erythematosus Unveil Activated Phosphoinositide 3-Kinase-δ Syndrome 2 in an Adult Patient
Source: Front Pediatr. 2021 Jul 8;9:702546. doi: 10.3389/fped.2021.702546 (PMC8295470; doi:10.3389/fped.2021.702546)

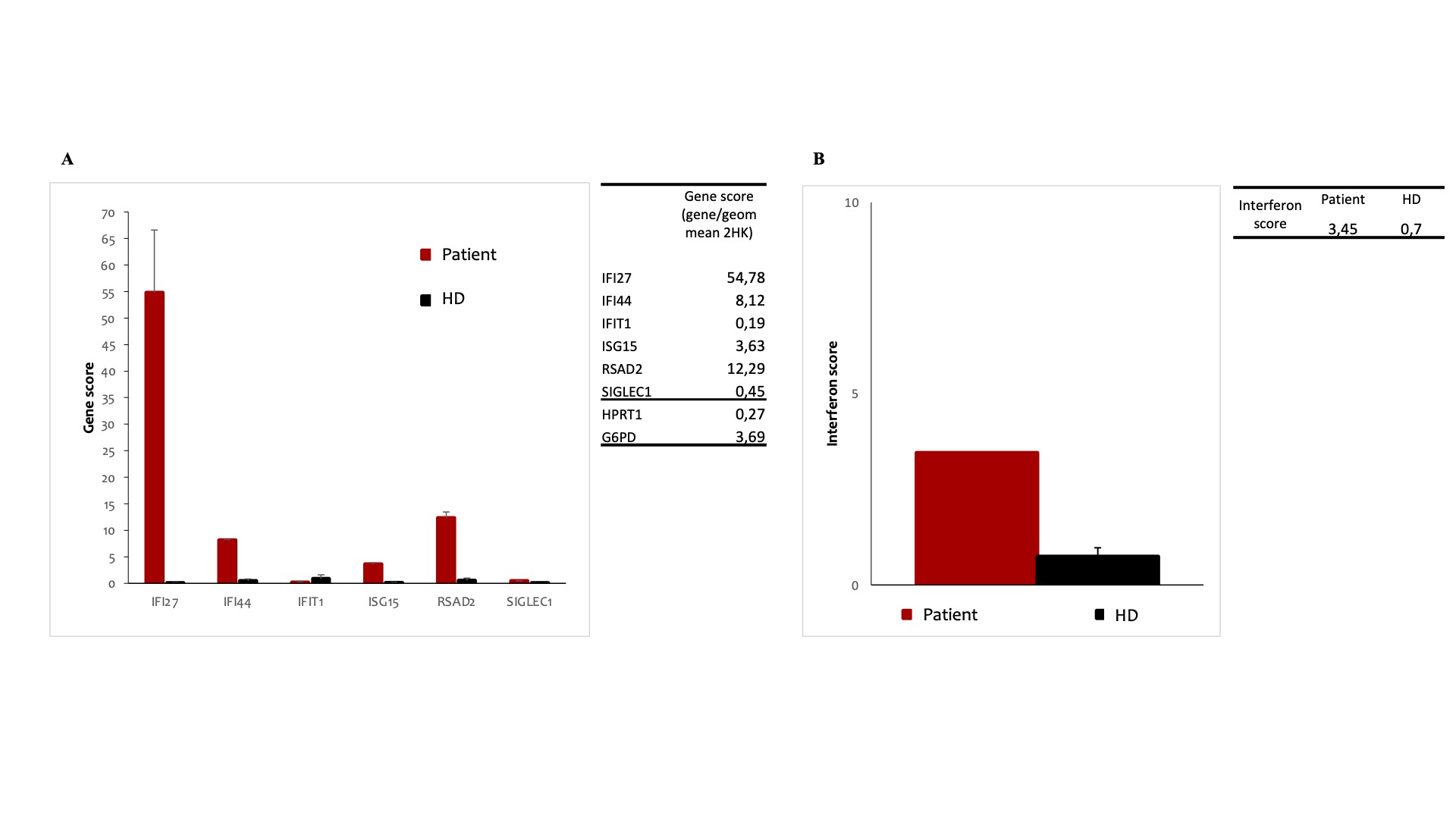

Supplement: Supplementary Figure 1 — Relative quantification of gene expression of six interferon-stimulated genes in peripheral blood. Serum IFN-I signature was increased as shown by (A) gene score and (B) interferon score. Interferon signature was obtained as published with modifications [J Clin Immunol. (2019) 39:476–85. doi: 10.1007/s10875-019-00645-0]. [file Image_1.JPEG]
